# Nature-inspired peptide of MtDef4 C-terminus tail enables protein delivery in mammalian cells

**DOI:** 10.1038/s41598-024-55274-4

**Published:** 2024-02-26

**Authors:** Lucia Adriana Lifshits, Yoav Breuer, Marina Sova, Sumit Gupta, Dar Kadosh, Evgeny Weinberg, Zvi Hayouka, Daniel Z. Bar, Maayan Gal

**Affiliations:** 1 Department of Oral Biology, Faculty of Medicine, The Goldschleger School of Dental Medicine, Tel Aviv University, 6997801 Tel Aviv, Israel; 2 BLAVATNIK CENTER for Drug Discovery, Tel-Aviv University, 6997801 Tel Aviv, Israel; 3 Institute of Biochemistry, Food Science and Nutrition, The Robert H. Smith Faculty of Agricultural, Food and Environment, The Hebrew University of Jerusalem, 76100 Rehovot, Israel

**Keywords:** Cell-penetrating peptide (CPP), Cellular uptake, Delivery agents, Peptides, Protein transduction, MtDef4, Therapeutics, Biochemistry, Peptides, Cell delivery, Peptide delivery, Protein delivery, Peptides

## Abstract

Cell-penetrating peptides show promise as versatile tools for intracellular delivery of therapeutic agents. Various peptides have originated from natural proteins with antimicrobial activity. We investigated the mammalian cell-penetrating properties of a 16-residue peptide with the sequence GRCRGFRRRCFCTTHC from the C-terminus tail of the Medicago *truncatula* defensin MtDef4. We evaluated the peptide’s ability to penetrate multiple cell types. Our results demonstrate that the peptide efficiently penetrates mammalian cells within minutes and at a micromolar concentration. Moreover, upon N-terminal fusion to the fluorescent protein GFP, the peptide efficiently delivers GFP into the cells. Despite its remarkable cellular permeability, the peptide has only a minor effect on cellular viability, making it a promising candidate for developing a cell-penetrating peptide with potential therapeutic applications.

## Introduction

Cell-penetrating peptides (CPPs) have emerged as powerful tools in biomedical research and therapeutics due to their ability to facilitate the efficient delivery of biomolecules through the cell membrane^1–3^. The latter selectively regulates the cellular entrance of ions and small molecules and is usually impermeable to larger peptides and proteins. However, the unique properties of CPP amino acid sequences enable them to overcome the cell membrane barrier, allowing for the intracellular delivery of therapeutic molecules, imaging agents, and nucleic acids. Consequently, the discovery and characterization of new CPPs can significantly advance the development of targeted drug delivery systems, gene therapies, and diagnostic approaches in a broad range of biotechnological fields^4–9^.

Over 1500 CPPs are currently listed on the CPP2.0 site^10,11^. Despite their highly divergent sequences and physicochemical properties, these peptides can be broadly categorized into cationic, lipophilic, or amphipathic sequences^12^. The mechanisms by which CPPs translocate into the cells are highly variable, primarily involving either endocytosis or direct permeation into the cytosol. The preference for these different internalization pathways is affected by membrane composition, peptide concentration, and specific biophysical properties^13^, all of which directly affect the cellular entry of the CPPs^14,15^. An additional classification criterion is the source of the peptide sequence, whether originating from a natural protein or synthetically designed^16,17^. Since the discovery of the transactivating transcription protein from HIV-1 (TAT peptide)^18^ and the penetration peptide from the Antennapedia homeotic transcription factor^19^, a variety of naturally derived CPPs have been characterized. Most of these are cationic peptides ranging from 6 to 30 amino acids in length^20–25^. Despite the large number of CPPs, significant drawbacks such as low penetration efficiency, poor stability, and immunogenicity often hinder the therapeutic and biotechnological applications of CPPs. Indeed, although various CPPs are in clinical trials, the Food and Drug Administration (FDA) has not yet approved CPPs for therapeutic use in humans. Thus, further discovery and characterization of novel sequences showing enhanced membrane permeability and stability with reduced immunogenicity and toxicity are of great interest.

Defensins, a family of compact, cysteine-rich antimicrobial peptides comprising approximately 50 amino acids, play a crucial role in fortifying vertebrates and plants immune system against various pathogens^26–28^. Owing to their antimicrobial attributes, plant defensins have attracted substantial interest due to their potential contributions to agriculture and medicine. Defensin antimicrobial characteristics position them as prospective candidates for devising natural alternatives to artificial pesticides. Furthermore, their proven efficacy against human pathogens places defensins as the basis for developing a therapeutic alternative for tackling antibiotic-resistant bacteria and fungal infections^29–31^.

**Figure 1:**
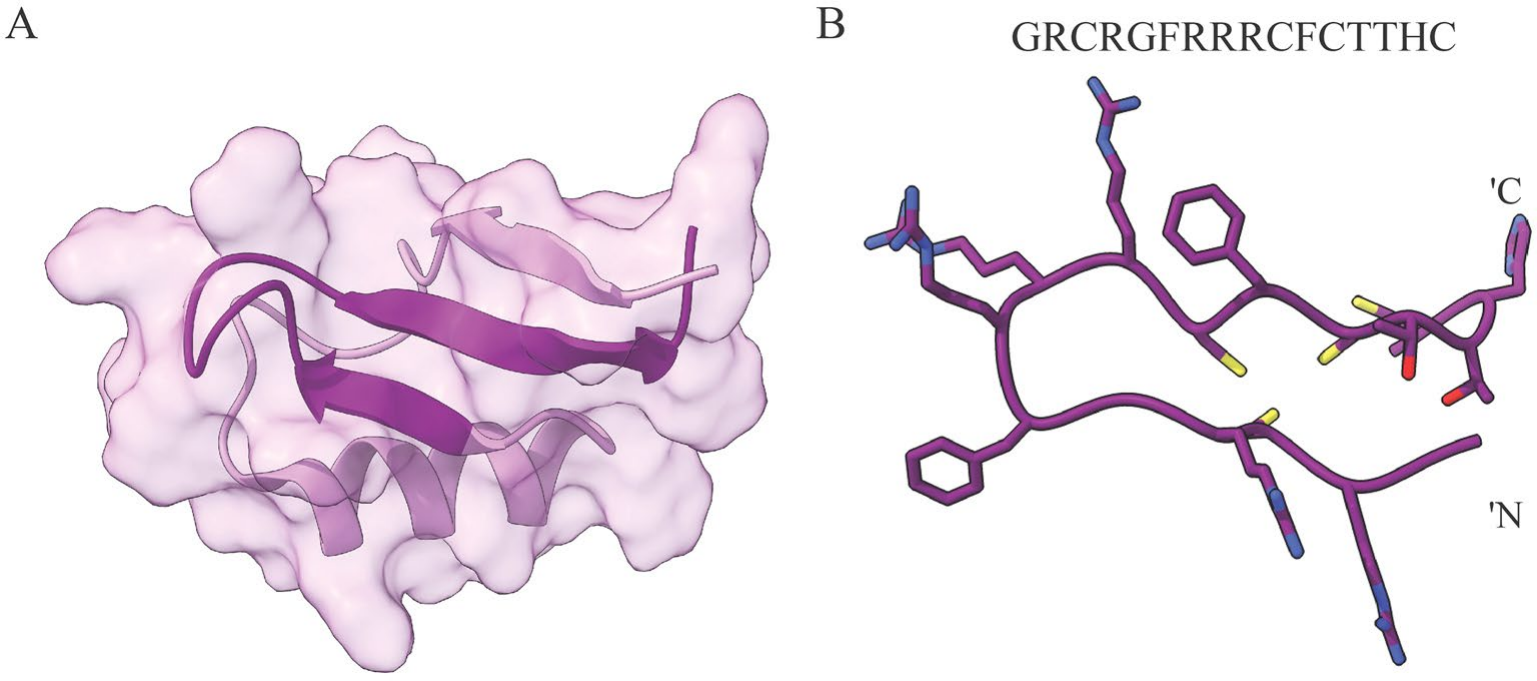
Structure of MtDef4 (PDB: 2LR3)^32^ and GMA4C peptide. (**A**) MtDef4 structure comprises 47 amino acids with a molecular weight of 5.3 kDa. The sixteen residues of the C-terminus tail GRCRGFRRRCFCTTHC (color-coded in dark purple) play a significant role in MtDef4’s function and cellular penetration. (**B**) The Alphafold2 predicted structure of GMA4C is represented by the purple color, while positive amino acids such as Arginine and Histidine are depicted in blue. Red and yellow are referred to Threonine and Cysteine, respectively.

The *Medicago truncatula* defensin 4 (MtDef4) belongs to the defensin protein family, known for inhibiting the growth of plant pathogens^32^ with a distinct activity profile against various fungal strains^33,34^. It is composed of 47 amino acids with a C-terminal tail that is characterized by the RGFRRR motif, pivotal for its antifungal effectiveness and ability to penetrate fungal cells (Fig. 1). Peptides derived from the C-terminal region of MtDef4 have exhibited potent antifungal properties^35^. These peptides are characterized by cationic sequences, enabling them to traverse cellular barriers and effectively penetrate fungal cells. The current study focuses on the 16-residue C-terminal peptide of MtDef4, known as GMA4C^35^, evaluating its penetration and ability to deliver proteins into mammalian cells. Our investigation reveals this peptide’s remarkable mammalian cell-penetrating characteristics and capability to deliver biomolecule cargo into the cells.

## Results

### Structural modeling of GMA4C

Figure 1 illustrates the structure of MtDef4, highlighting the region containing the 16-residue tail with the sequence GRCRGFRRRCFCTTHC (GMA4C, depicted in dark purple). These residues assemble into a scorpion toxin-like domain, featuring two cysteine-rich beta strands interconnected by a positively charged loop^32^. Figure 1B depicts the predicted structure of the peptide in its free form. To evaluate GMA4C’s ability to deliver proteins into mammalian cells, it was additionally fused either as an N- or C-terminus tag to green fluorescent protein (GFP). Figure S1 illustrates the architecture of these constructs (Fig. S1A) and presents structural models of the fused proteins (Fig. S1B).

### Evaluation of GMA4C effect on cellular viability

**Figure 2:**
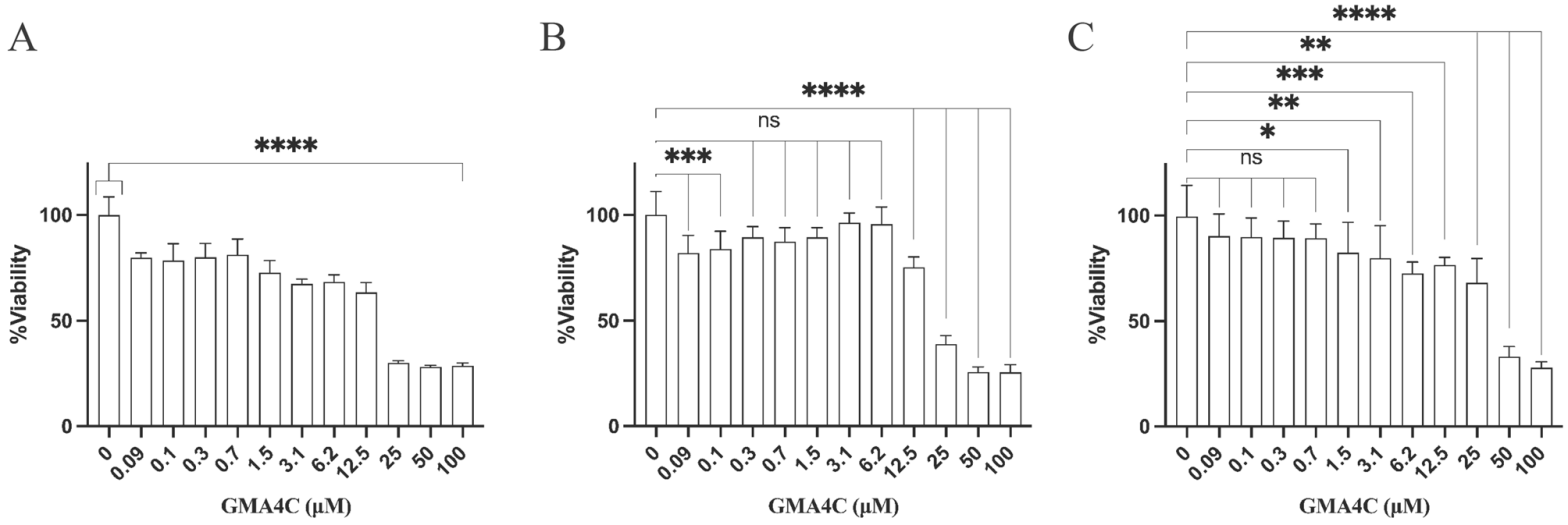
GMA4C effect on the cellular viability. Bar plot showing the percentage of viable cells vs. GMA4C peptide concentrations. (**A**) HeLa (**B**) HGF and (**C**) C2C12 cells were seeded in a 96-well plate at 2,500 cells/well and incubated with variable concentrations of GMA4C peptide. Viable cells were quantified by methylene blue following 48 h. Values are shown as mean ± SD of four replicates. *p < 0.05, **p < 0.005, ***p < 0.0005, ****p < 0.0001.

CPPs must show relatively low toxicity to be considered for any biomedical application. Before investigating the ability of the peptide containing the 16-residue C-terminal tail of MtDef4 (GMA4C) to penetrate mammalian cells and facilitate the delivery of larger proteins, we assessed its impact on cellular viability, establishing a safe and effective concentration range for in-cell applications. To this end, HeLa, HGF, and C2C12 cells were incubated with varying concentrations of the GMA4C peptide for 48 h. Cellular viability and proliferation were assessed using the Methylene Blue assay (Fig. 2), resulting in an IC50 value of ca. 20 µM for the Hela and HGF and 35 µM for the C2C12 cells. Thus, the peptide significantly affects cell viability only in the tens of micromolar range, establishing safe working concentrations for further investigations.

### GMA4C penetration into mammalian cells

**Figure 3:**
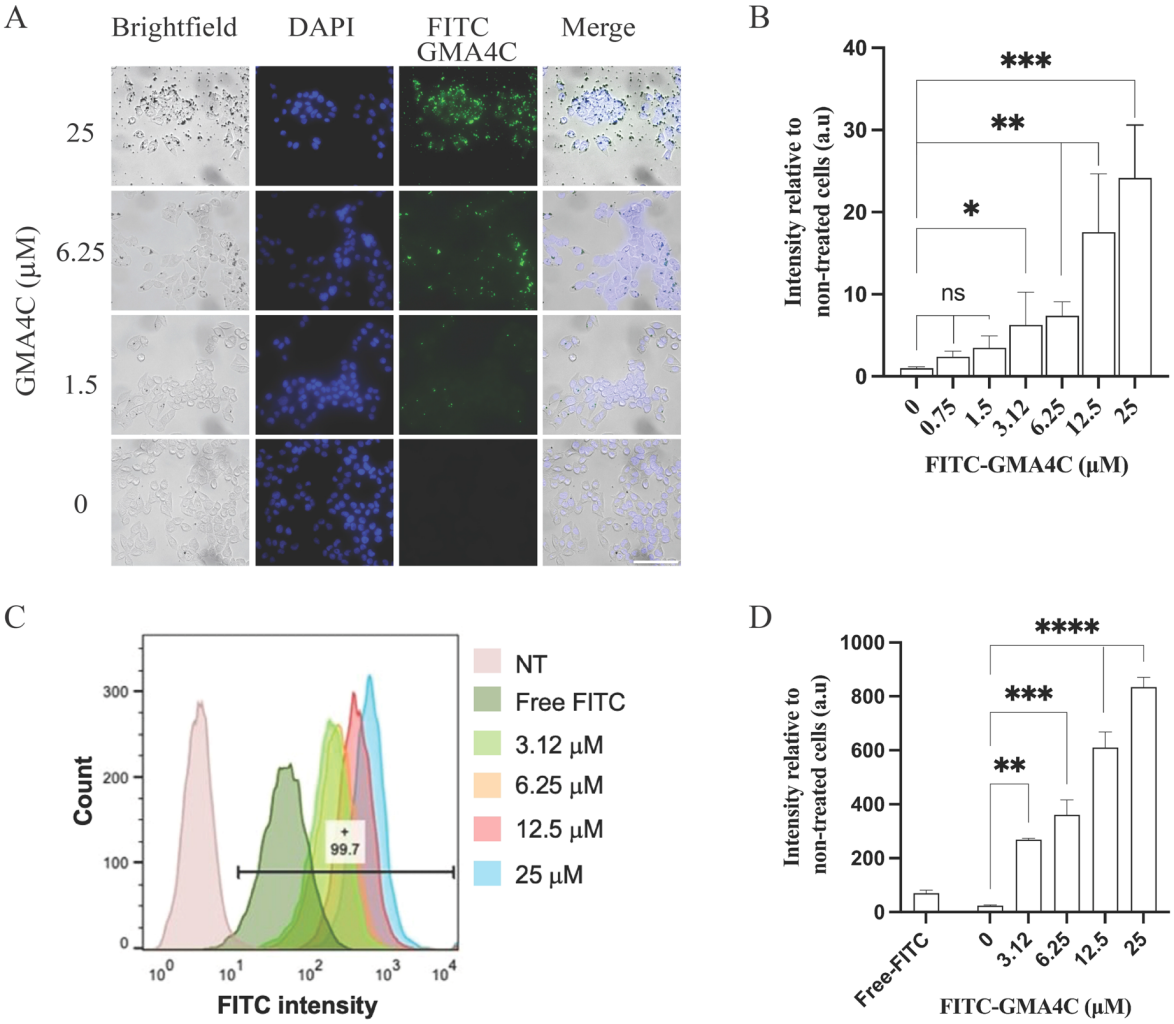
Characterization of GMA4C penetration into HeLa cells by confocal imaging and flow cytometry analysis. (**A**) Representative confocal images of HeLa cells treated with variable concentrations of FITC-GMA4C. Cells were incubated with indicated FITC-GMA4C concentrations for two hours at 37 °C, followed by PBS washing and subsequent fixation with 4% PFA and post-fixation staining with DAPI. Images of cells were taken by confocal microscopy, visualizing FITC-GMA4C (green) and DAPI (Blue). Scale bar: 100 µm. (**B**) Quantification of FITC-GMA4C fluorescence intensity. Bars show the mean green fluorescence intensity relative to non-treated cells and normalized to the number of cells. Values were measured using the Color Threshold tab in Fiji. Values shown are the mean ± SD of three replicates. ns—non-significance, *p < 0.05, **p < 0.01, ***p < 0.005. (**C**) Quantification of FITC-GMA4C peptide uptake by cells. HeLa cells were incubated with variable FITC-GMA4C peptide concentrations for 2 h, washed, treated with trypsin (0.25%) and subjected to flow cytometry. Diagram representing the uptake of the FITC-containing cells based on the flow cytometry analysis of three independent experiments. Free FITC concentration—25 µM. The horizontal bar and number indicate the percentage of gated FITC-positive cells treated with 25 µM of FITC-GMA4C. NT-non-treated. (**D**) Quantitative evaluation of flow cytometry signal intensity. Values were measured using FloJo flow cytometry software. Mean and SD values are calculated from three replicates. **p < 0.005, ***p < 0.0005, ****p < 0.0001.

To evaluate the capability of GMA4C to penetrate mammalian cells, we synthesized the peptide with a fluorescein isothiocyanate (FITC) fluorescent probe at its N-terminus (FITC-GMA4C). Subsequently, we incubated HeLa cells with varying concentrations of FITC-GMA4C for two hours. After thorough washing, we visualized the cells using confocal microscopy imaging. Remarkably, we observed the accumulation and intracellular localization of FITC-GMA4C within the cells, even at low micromolar concentrations (Figs. 3A,B, and S7A). The entry of FITC-GMA4C is depicted by green dots within the cells, indicating effective peptide transduction. To evaluate the generality of this peptide, we repeated these experiments with additional cell lines that differ in their membrane composition. Notably, FITC-GMA4C also demonstrates similar cell-penetrating capabilities in all other cell types tested, including human gingival fibroblast cells, C2C12 myoblast cells, and fungal strain *A. flavus* (Figs. S2–S4 respectively). To validate the cellular penetration of FITC-GMA4C further, we conducted flow cytometry analysis on HeLa cells following their incubation with varying concentrations of FITC-GMA4C (Fig. 3C,D). To ensure that the detected fluorescence signal originated only from peptides internalized within the cells and not from peptides bound to the cell membrane, we washed the cells and subjected them to trypsin treatment at 37 °C. This step efficiently eliminates specific and nonspecific extracellular membrane-associated peptides. While the free-FITC control only slightly accumulated in the cell, the FITC-labeled peptide significantly shifted the entire cell population in a concentration-dependent manner.

**Figure 4:**
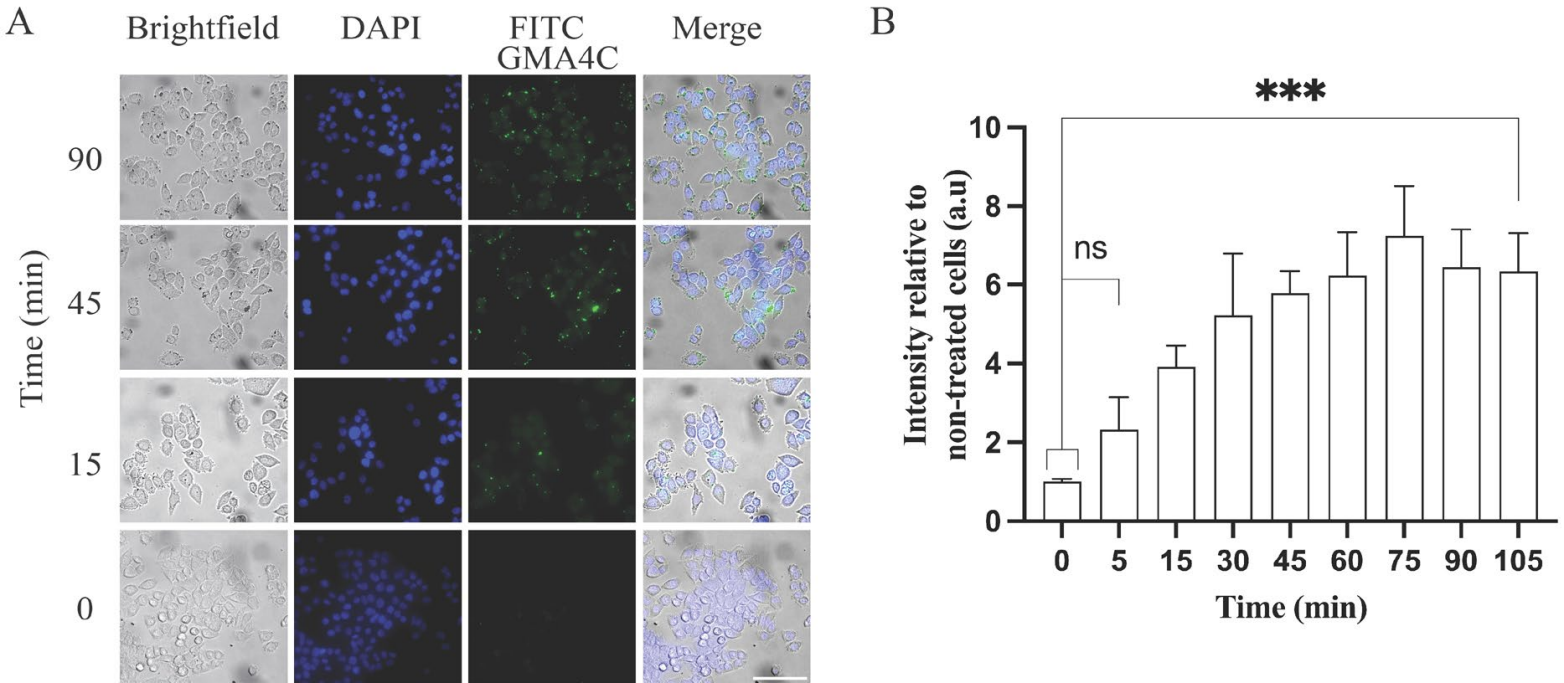
Time response of FITC-GMA4C uptake into HeLa cells. (**A**) Cells were cultured with 6 µM of FITC-GMA4C at 37 °C, followed by PBS washing and subsequent fixation with 4% PFA and post-fixation staining with DAPI. Images of cells were taken by confocal microscopy visualizing FITC-GMA4C (green) and DAPI (Blue). Scale bar: 100 µm. (**B**) Quantification of FITC-GMA4C fluorescence intensity. Bars show the mean green fluorescence intensity relative to non-treated cells and normalized to the number of cells. Values were measured using the Color Threshold tab in Fiji. Values are shown as mean ± SD of three replicates.

CPPs that demonstrate rapid internalization are preferable for biomedical use, as this enables a quicker response time while reducing their dwell time in the extracellular environment, which may modify or degrade them and reduce the risk of an immunological reaction. To this end, we explored the internalization kinetics of FITC-GMA4C into the cells. At the outset, we performed a dose–response investigation using a twofold dilution, identifying optimal conditions at 6uM for peptide visualization (Figs. 2 and 3). Subsequently, HeLa cells were cultured with 6 µM FITC-GMA4C and imaged at different time points. A discernible increase in the fluorescence signal, indicative of peptide uptake, becomes apparent within a few minutes, with a t_1/2_ of ~ 15 min (Fig. 4).

### GMA4C fusion facilitates GFP delivery into mammalian cells

**Figure 5:**
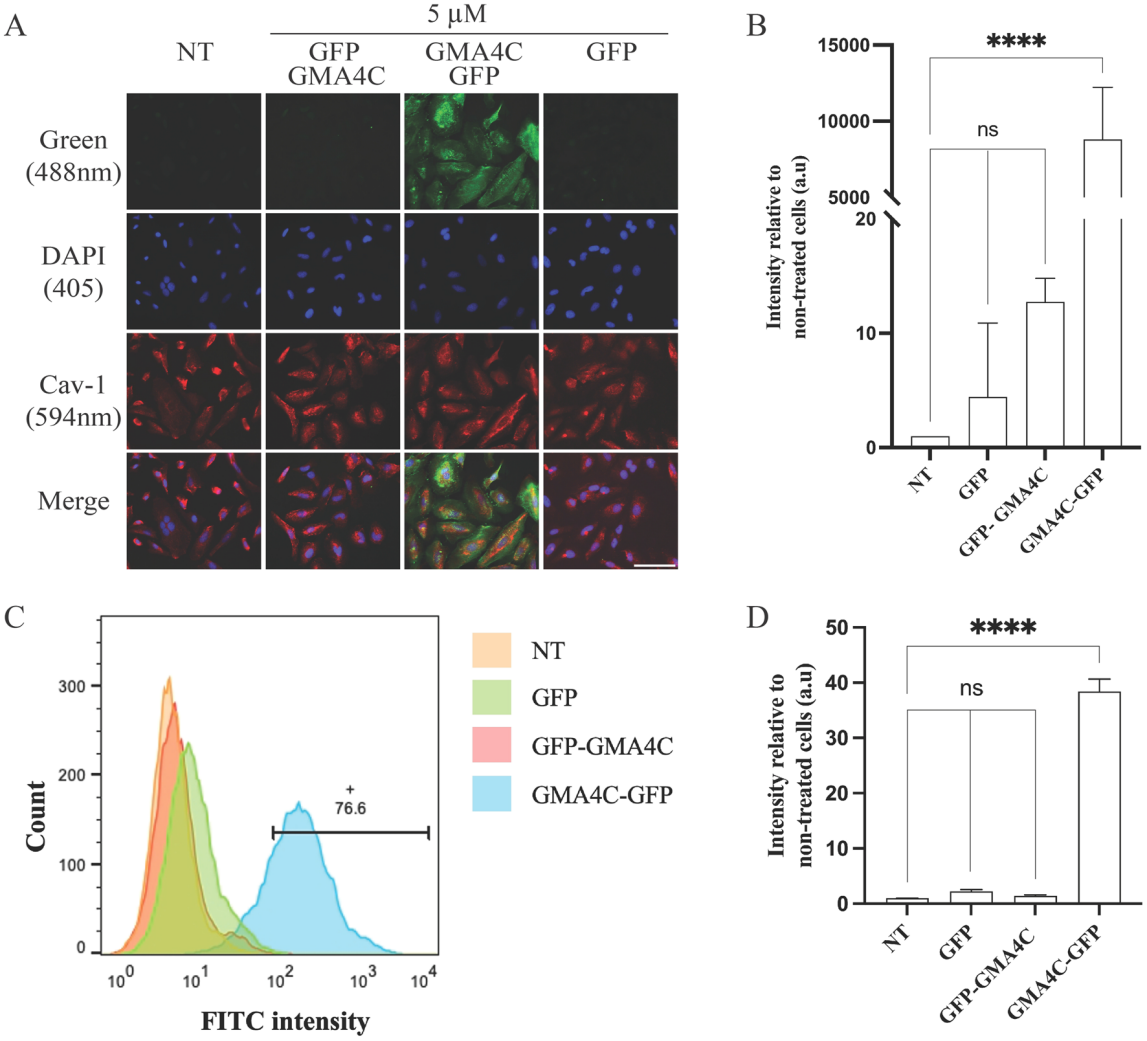
Cellular delivery of GFP conjugated to GMA4C peptide. (**A**) HeLa cells were incubated with the recombinant proteins GFP, GMA4C-GFP and GFP-GMA4C at a concentration of 5 μM for 2 h. Images of cells were taken by confocal microscopy visualizing DAPI (blue), GFP (green) and Caveolin-1 (red) channels. Scale bar: 100 µm. (**B**) Quantification of GFP fluorescence intensity. Bars show the mean fluorescence intensity relative to non-treated cells and normalized to the number of cells. Values were measured using the Color Threshold tab in Fiji, shown as mean ± SD of three independent replicates. ns—non-significance, ****p < 0.0001. (**C**) Flow cytometry analysis of the cellular uptake of GFP, GMA4C-GFP and GFP-GMA4C to HeLa cells. HeLa cells were treated with 5 μM of the recombinant proteins for 2 h. Diagram representing the uptake of the FITC-containing cells based on the flow cytometry analysis of three independent experiments. NT—Non-treated. The horizontal bar and number indicate the percentage of gated FITC-positive cells. (**D**) Quantitative evaluation of flow cytometry signal intensity. Values were measured using FlowJo cytometry software. Mean and SD values are calculated from three replicates. Values were measured using the Color Threshold tab in Fiji, shown as mean ± SD of three replicates. ns—non-significance, ****p < 0.0001.

Given the remarkable rate and efficiency of the free peptide cellular penetration, we further investigated its capacity to enhance the delivery of large biomolecules into cells. For that purpose, we evaluated the cellular permeabilization of recombinant GFP, expressed and purified with an N- or C-terminus fusion of GMA4C: GFP, GMA4C-GFP and GFP-GMA4C, respectively (Fig. S1). As expected, GFP alone showed no ability to penetrate cells (Fig. 5A). Surprisingly, while GFP-GMA4C minimally penetrated the cells, GMA4C-GFP penetrated the cells exceptionally well (Figs. 5A,B and S6). This is despite structural predictions indicating that the GMA4C is accessible in the GFP’s N- and C-terminus (Fig. S1B). To gain further insights into the potential cellular mechanisms responsible for GMA4C transduction, we performed staining with a caveolin-1 (Cav-1) antibody. The GMA4C-GFP protein, visualized in the green channel, demonstrates partial co-localization with Cav-1 in the red channel. This observation suggests a localization of part of the GMA4C-GFP within endosomes, possibly due to endocytosis mediated by a caveolin-dependent pathway in various cell lines (refer to Figs. 5A and S3). The partial co-localization implies that additional cellular entry mechanisms may be involved^36,37^. Subsequently, we quantified the cellular uptake of the exogenous proteins using flow cytometry analysis. Cells were incubated with each recombinant protein at a concentration of 5 μM for 2 h. The flow cytometry data further supported our initial observation that GMA4C-GFP exhibits enhanced cellular uptake compared to GFP-GMA4C and GFP alone (Fig. 5C,D). Since fixation of the cells could impact the permeability and distribution of CPPs in the cell^38,39^, we assessed the GMA4C-GFP cellular entry via live-cell imaging. Figure S7 shows confocal images of HeLa cells treated with GMA4C-GFP without fixation, further revealing an efficient protein transduction.

### Low temperature reduces GMA4C cellular permeability

**Figure 6:**
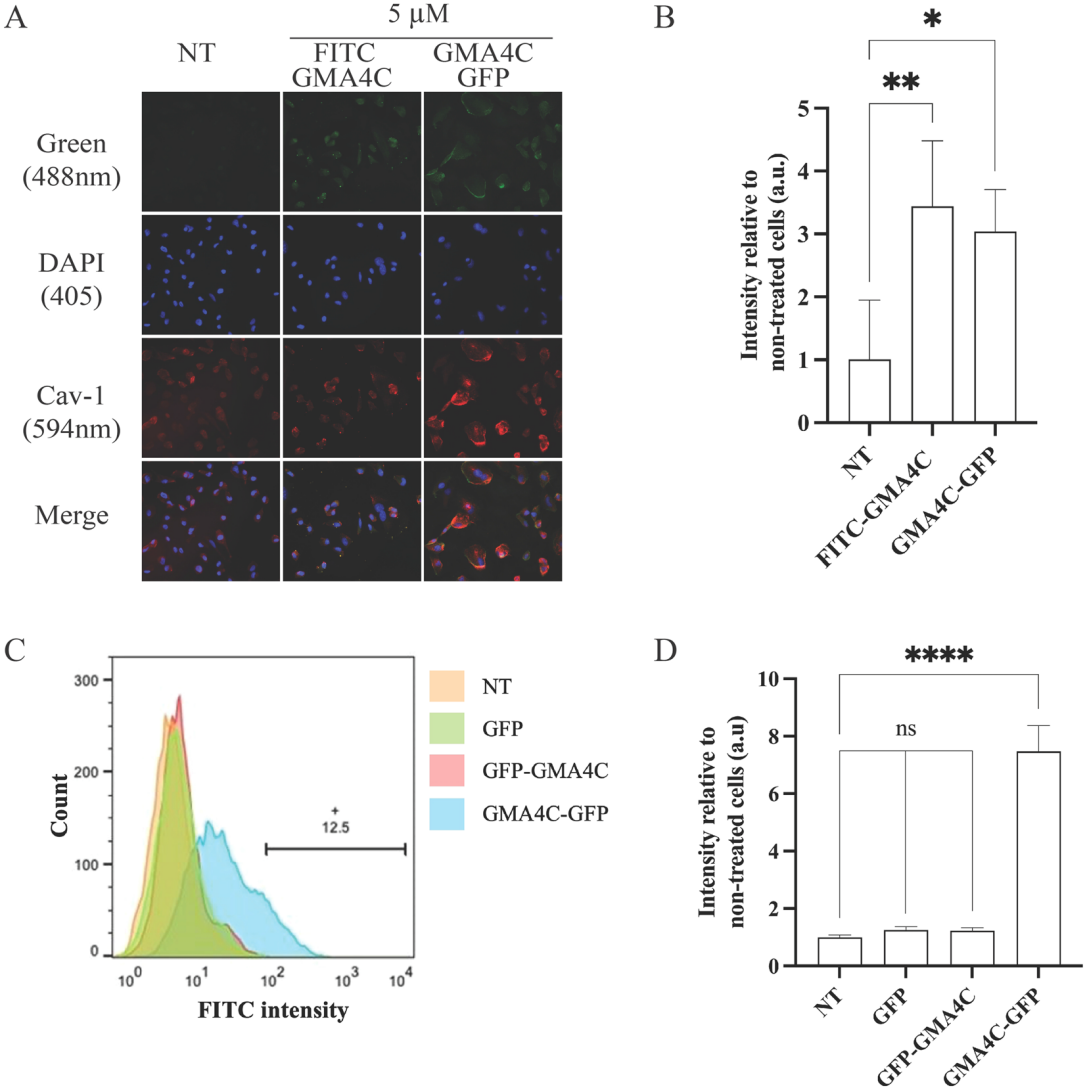
Low-temperature effect on the cellular uptake of GFP conjugated to GMA4C in HeLa cells. (**A**) Representative confocal images of HeLa cells incubated with 5 μM of FITC-GMA4C and GMA4C-GFP for 2 h at 4 °C. Images of cells were taken by confocal microscopy, visualizing FITC or GFP (green), DAPI (Blue) and Cav-1 (red). Scale bar: 100 µm. (**B**) Quantification of confocal images signal intensity. Bars show the mean green fluorescence intensity relative to non-treated cells and normalized to the number of cells. Values were measured using the Color Threshold tab in Fiji. Values show the mean ± SD of three replicates. ns—non-significance, *p < 0.012, **p < 0.0026. (**C**) Flow cytometry analysis of the cellular uptake of FITC-GMA4C and GMA4C-GFP to HeLa cells incubated with 5 μM of the recombinant proteins for 2 h at 4 °C. Diagram representing the uptake of the FITC or GFP-containing cells based on the flow cytometry analysis of three independent experiments. NT—Non-treated. The horizontal bar and number indicate the percentage of gated GFP-positive cells. (**D**) Quantitative evaluation of flow cytometry signal intensity. Values were measured using FloJo flow cytometry software. Mean and SD values are calculated from three replicates. ns—non-significance, ****p < 0.0001.

At low temperatures, membrane lipids undergo a reversible change to a rigid structure, thus limiting energy-dependent endocytosis. Consequently, active endocytosis is anticipated to result in a lower cellular permeabilization. To delve deeper into the penetration mechanism of GMA4C through the cell membrane, cells were supplemented with the proteins at low-temperature conditions (4 °C) for 2 h and examined using confocal microscopy. A significant reduction in the penetration of FITC-GMA4C and GMA4C-GFP to HeLa cells at 4 °C is observed (Fig. 6A,B). Flow cytometry further supports these findings, showing a reduced penetration efficiency at 4 °C. Indeed, a reduced percentage of GFP-positive cells was observed at 4 °C (Figs. 6C,D and S5) compared to 37 °C (Fig. 5C).

## Discussion

CPPs have gained significant attention for their unique ability to facilitate the delivery of various cargoes across cellular membranes and various CPPs have been investigatedic for their effectiveness in delivering large molecules. Typically, most short CPPs are recognized by their positive charge, allowing them to traverse the cell membrane at relatively low concentrations. Although several clinical trials involving conjugated CPPs are ongoing, no CPP-based drug has been approved^40,41^. Consequently, the discovery and design of novel CPPs with improved efficiency, specificity, and safety profiles remain highly desirable in biomedical research. Intriguingly, natural antimicrobial peptides, with their innate cationic and membrane-penetrating attributes, emerge as particularly promising candidates for CPP development. These naturally occurring peptides could potentially serve as blueprints for designing CPPs that not only achieve enhanced intracellular delivery but also set new benchmarks in specificity and safety.

This study investigated the cell-penetrating properties of a 16-residue peptide derived from the C-terminus tail of the antifungal plant protein MtDef4. To characterize GMA4C’s cellular penetration capabilities, we employed a combination of confocal imaging, FACS and western blotting (Figs. 5, 6, and S7), demonstrating its remarkable efficiency in penetrating cells. Our results reveal that this peptide can penetrate not only HeLa cells but also other cell types, such as HGF and C2C12 (Figs. S2 and S3). Additionally, we observed penetration into the cell wall of *A. flavus* (Fig. S4), which aligns with recent findings in other fungal cells^35^.

The capacity of CPPs to deliver large biomolecules into mammalian cells is of paramount importance. To assess the effectiveness of GMA4C in this regard, we utilized *E. coli* to express a recombinant GFP protein linked with an N-terminus GMA4C peptide. The CPP fusion enabled efficient cellular penetration into cells despite being ~ 14 times larger than GMA4C (29.7 kDa including His-tag vs. 2.09 kDa). However, we observed limited cellular transduction when the fusion was made to the C-terminus of GFP (Fig. 5). While the exact mechanism hindering the latter’s cellular entry remains unclear, it may be attributed to structural disparities or the masking of functional segments of GMA4C, thereby impeding its efficient penetration. Notably, structural predictions by AlphaFold suggest that GMA4C remains exposed in both structures. Thus, it could be that excited state population or structural differences that arise only upon contact with the cell membrane drive penetration differences between the two proteins. We also note that the predicted structure of GMA4C when fused to GFP (Fig. S1) differs from its resolved structure within MtDef4 protein (Fig. 1). We speculate that the closer spatial proximity of GMA4C to the bulk globular structure is essential for an efficient transport across the membrane in GMA4C-GFP. By contrast, the residual amino acids of the GFP C-terminus protrude outside the globular structure, distance GMA4C from the globular structure, and preclude efficient transport. However, the differential cellular permeability of the two constructs may be attributed to the proximity of the His-tag, facilitating the cellular entry^42^. An additional important aspect involves the formation of disulfide bonds, which could affect the penetration properties of GMA4C. Intriguingly, the distance between Cys residues in the peptide in its free form seems not to support the formation of disulfide bonds (Fig. 1B). Also, the structural model of the peptide fused to GFP (Fig. S1B) suggests that the distances between cysteine residues are too large for the formation of a disulfide bond. Therefore, additional structural research is required to elucidate the precise structural orientation of GMA4C when fused to a cargo protein and its consequent impact on cellular penetration. In addition, if the peptide lacks disulfide bonds, imparting it by synthetic approaches could further improve its cellular permeability.

Minimal effect on cell viability is a prerequisite for CPPs considered for most biomedical applications. Our results indicate that efficient cellular penetration of GMA4C occurs within a single micromolar concentration range, much lower than its cellular viability IC50. This implies that the potential application of the peptide can be at a concentration approximately an order of magnitude lower than its toxic range. Moreover, GMA4C has a fast penetration time, where significant uptake is observed under 15 min and an equilibrium is reached within 30 min, and nearly 100% penetration was quantified into the cells when supplemented with 10 µM of GMA4C-GFP (Fig. S6). It is enlightening to compare GMA4C to prototypical CPPs such as TAT and multi-arginine R9. Generally, the effectiveness of CPPs varies a lot based on the experiment, the cargo protein, and the type of cells used. Multiple studies have shown that TAT and R9, as well as additional peptides such as penetratin and cupid, exhibit a high efficiency of 70–90% in delivering diverse cargo molecules into cells^43,44^. Our study, which shows the first evaluation of GMA4C as a CPP in mammalian cells, revealed a high cell-penetration efficiency of > 90% in delivering GFP into the cells. Also, in terms of viability, similarly to TAT and R9, GMA4C shows no acute cellular toxicity, thus making its application amendable even at relatively high concentrations. In addition, supported by previous studies^35^, we showed the ability of GMA4C to penetrate fungal cells (Fig. S4). The latter are generally known for their restrictive cell walls that block the passage of proteins and peptides. The majority of CPPs, which have been assessed in mammalian cells, have not undergone investigation in fungal cells. However, TAT and Cupid have successfully shown the ability to penetrate *S. cerevisiae* and *C. albicans,* respectively^45,46^.

While our study primarily focused on the efficacy of cellular penetration, the precise cellular mechanism by which GMA4C enters cells remains unclear. Indeed, determining an exact cellular uptake mechanism of CPPs is often challenging^44,47^. In the current study, we observed that the cellular penetration of FITC-GMA4C and GMA4C-GFP is temperature-dependent, with reduced efficiency at lower temperatures (Figs. 4, 5, and S5). This suggests that the cellular entry is an energy-dependent process, different from what was reported on Arg-rich CPPs such as TAT^48^. The flow cytometry findings are in qualitative accordance with the observations from confocal microscopy, indicating that the GMA4C peptide can deliver and translocate into diverse cell types and could serve as a possible generalizability as CPP. Moreover, GMA4C-GFP is partially co-localized with the endosomal marker Cav-1, suggesting that cellular entry of the CPP could be driven by endocytosis rather than direct membrane translocation^49^. However, we can’t rule out the possibility of additional factors preventing GMA4C cellular entry at low temperatures, such as the cell membranes becoming less fluid and more rigid. This could hinder the entry of CPPs attempting to cross the membrane under such conditions. Nonetheless, partial protein permeability persisted despite the reduced activity of endocytosis, indicating the possibility of peptide diffusion through an energy-free process. In addition, temperature-dependent structural differences of the protein or peptide at high and low temperatures could affect its functionality.

To assess the peptide’s suitability for clinical use, conducting in vivo safety studies is essential. These studies should focus on its performance, considering factors like circulation complexities, degradation, and hemolytic properties of the peptide. Adjustments, such as increasing concentration for optimal in-vivo penetration or structural modifications for enhanced stability and safety, might be necessary. Additionally, the potential immunogenic effects associated with CPPs must be evaluated.

## Methods

### Peptide synthesis

GMA4C peptide with and without FITC (C21H11NO5S) was synthesized by Peptide2.0 with 90% purity (Chantilly VA, United States). The peptide was dissolved in DMSO to a stock concentration of 50 mM.

### Cloning of GFP-GMA4C and GMA4C-GFP plasmids

Primers for PCR are listed in Table S1. Cloning of GMA4C sequence to pET plasmid containing an N-terminus Hisx6 tag and GFP (Addgene # 29663) was done by whole plasmid amplification using the indicated forward and reverse primers for GFP-GMA4C (primers 1 and 2) and GMA4C-GFP (primers 3 and 4). Following digestion at 37 °C with DpnI, the plasmids were phosphorylated by T4 Polynucleotide Kinase and ligated by T4 Ligase (NEB).

### Expression of recombinant proteins

Expression of recombinant proteins (GFP, GFP-GMA4C and GMA4C-GFP) was performed by the transformation of the plasmid into *E. coli* BL21 (DE3). Bacterial growth was carried out at 37 °C and 200 rpm until OD_600_ was ~ 0.8, and protein expression was induced by supplementing 1 mM Isopropyl β-d-1-thiogalactopyranoside (IPTG) for 16 h at 25 °C. Following three sonication cycles of three minutes each, the bacterial lysate was centrifuged at 16,000xg for 30 min. The supernatant was passed onto a nickel column, and after extensive washing, the protein was eluted with 300 mM imidazole. Following buffer exchange to phosphate-buffered saline (PBS), the protein was kept frozen with 50% glycerol. Protein concentrations were assessed using NanoDrop (NanoDrop OneC), and the purity of all proteins was estimated to be higher than 90% based on SDS gel.

### Cell cultures

Experiments with primary human gingival fibroblasts (hGFs) were approved by the Tel Aviv University institutional review board (IEC No. 0001006-1). Informed consent was obtained from all patients and all research was performed in accordance with relevant guidelines and regulations. Human gingival fibroblasts (HGF) derived from masticatory mucosa were cultured as previously described^50,51^. Briefly, HGF cells were thawed with the appropriate medium and the third passage was examined. Cells were grown on a Alpha modified Eagle’s minimum essential medium (α-MEM) with 10% fetal calf serum, 100 µ/mL penicillin, and 100 mg/mL streptomycin, 1% Sodium Pyruvate. The cervical cancer cell line HeLa and C2C12 myoblast cells were a gift from Lihi Adler-Abramovich lab, Tel Aviv University, and cultured in a standard culture medium (Dulbecco’s modified Eagle’s medium (DMEM)) with 10% fetal calf serum, 2 mM glutamine, 100 µ/mL penicillin, and 10 µg/mL streptomycin.

### Immunofluorescence confocal microscopy

For microscopy images, HeLa and C2C12 cells were cultured the day prior to the experiment on round-glass coverslips in a 24-well plate or a 96-well glass bottom plate and incubated with medium supplemented with the indicated concentrations of peptide/protein at 37 °C under 5% CO_2_. Cells were washed three times with cold PBS, followed by a cleansing process utilizing light shaking with three washes of PBS, then three washes with 25 mM of Hepes to eliminate nonspecific protein binding. The cells were fixed with 4% paraformaldehyde (PFA) in PBS for 20 min and washed three times with PBS to remove the PFA. All samples were stained with 1 mg/mL 4′,6-diamidino-2-phenylindole (DAPI Staining Solution ab228549, Abcam). Cav-1 (ab2910, Abcam), β-tubulin (05-661, Merck). Images were taken directly from the 96-well plate glass bottom, while coverslips from the 24-well plate were transferred into a glass slide and placed on the plate with sodium phosphate buffer mounting medium (25% Mowiol, 50% glycerol, 25% sodium base pH 8.0). Images were taken using a ZEISS LSM 900 in confocal mode, utilizing Zen 3.1 software. Fluorescence readouts were obtained for blue, green, bright red and far-red at ex/em of 358/461 nm, 495/519 nm, 561/594 nm, and 652/668 nm, respectively.

### Image analysis

Image acquisition and analysis were done with Fiji/ImageJ (version 2.14.0/1.54f), https://imagej.net/software/fiji/downloads. The fluorescence intensity of FITC-labeled peptide/recombinant protein was quantified using the color threshold option, which monitored the fluorescence values and normalized them by cell number.

### Cellular viability

HeLa, HGF, and C2C12 cells were seeded at 2.5x × 10^4^ per well in 96 well plates and cultured overnight. Then, cells were treated with variable concentrations of GMA4C peptide and incubated for 48 h. Cells were fixed with 4% paraformaldehyde (PFA) for 1 h at 25 °C, washed once with 0.1 M borate buffer pH 8.5, and stained with 1% methylene blue in 0.1 M borate buffer for 30 min. Excess of color washed and color was extracted by adding 0.1 M HCl (1 h, 25 °C) and read on a plate reader (SYNERGY H1, BioTek) at a wavelength of 595 nm.

### Structural modeling and visualization

GFP, GMA4C-GFP, GFP-GMA4C protein structure predicted by AlphaFold2 with MMseqs2 (https://colab.research.google.com/github/sokrypton/ColabFold/blob/main/AlphaFold2.ipynb). Structural visualization of GFP proteins and *Medicago truncatula* (PDB ID: 2LR3) was performed using UCSF ChimeraX^52^.

### Flow cytometry

Cells were seeded at a density of 3 × 10^5^ cells per well in a 6-well plate and grown at 37 °C in a 5% CO2 atmosphere in DMEM supplemented with 10% (v/v) FBS for 18 h. Cells were then incubated with indicated concentrations of peptide/proteins in fresh DMEM at 37 °C or 4 °C for 2 h, followed by washing three times with PBS. Cells were treated with Trypsin (100 μl/well of 0.25%). 1 ml PBS was added to the wells with the trypsinized cells, centrifuged, the supernatant was discarded, and the cells were resuspended in cold PBS with 2.5% FBS and analyzed by flow cytometry. FITC/GFP signal was monitored using Flow cytometer S1000EXi (Stratedigm, San Jose, CA). 10,000 cells were analyzed per single read. Dead cells were gated out from the analysis. Data were analyzed by CellCapTure and FlowJo v10.

### Western blotting

To determine whether the GFP tagged proteins internalized into HeLa cells, cells were cultured for two hours, washed with PBS, scraped with Buffer H (HEPES 20 mM, KCl 20 mM, MgCl_2_ 2 mM, EDTA 1 mM, EGTA 1 mM) containing a 1:200 dilution of Protease inhibitor (cat. 539134, Merck) and sonicated twice. The resulting cell lysate was mixed with a 4 × sample buffer and boiled at 95 ℃. Equal amounts of total protein were loaded on 10% SDS-PAGE gel and transferred to nitrocellulose membranes that were further blocked with 5% skim milk and incubated overnight with primary antibodies toward GFP (cat. ab6877, Abcam), Ku70 (cat. sc-5309, Santa Cruz Biotechnology), β-Tubulin (cat. AB16901, Merck). Protein marker PM2500 was used (SMOBIO, A2S). After extensive washing with tris-buffered saline supplemented with 0.1% Tween-20 (TBST), the membrane was further incubated with an HRP-conjugated secondary antibody for 1 h. Chemiluminescence values were read following the addition of ECL substrate (cat. AP124P, Merck) in a ChemiDoc imager (Bio-Rad, CA).

### Statistical analysis

Data analysis, bar plots, and statistical evaluations were performed using GraphPad Prism version 10.1.1 (www.graphpad.com). One-way ANOVA followed by Dunnett’s multiple comparisons post hoc test.

### Supplementary Information

Supplementary Information.

## Data Availability

All data generated and analyzed during this study are included in the manuscript and its supplementary Information files.
