# Supplementary material for: Nature-inspired peptide of MtDef4 C-terminus tail enables protein delivery in mammalian cells
Source: Sci Rep. 2024 Feb 26;14:4604. doi: 10.1038/s41598-024-55274-4 (PMC10897151; doi:10.1038/s41598-024-55274-4)
Supplement: Supplementary file 1 — Supplementary Information. [file 41598_2024_55274_MOESM1_ESM.pdf]

Supplementary information

Nature-Inspired Peptide of MtDef4 C-terminus Tail Enables Protein Delivery in Mammalian Cells

Lucia Adriana Lifshits[1], Yoav Breuer[1], Marina Sova[1], Sumit Gupta[1], Dar Kadosh[1], Evgeny Weinberg[1], Zvi Hayouka[2], Daniel Z. Bar[1] and Maayan Gal[1]

[1]Department of Oral Biology, The Goldschleger School of Dental Medicine, Faculty of Medicine, Tel Aviv University, Tel Aviv 6997801, Israel

[2]Institute of Biochemistry, Food Science and Nutrition, The Robert H. Smith Faculty of Agricultural, Food & Environment, The Hebrew University of Jerusalem, Rehovot 76100, Israel

Corresponding author

mayyanga@tauex.tau.ac.il; Tel.: +972-50-7987058

Supplementary Table S1

**Primer name**	**Sequence**	**Plasmid**
Primer 1	F: 5'-CGTCGCCGCTGCTTCTGCACCACGCATTGTTAACGGATCCGAATTCGAG-3'	GFP-GMA4C
Primer 2	R: 5'-AAAACCACGGCAACGGCCACTGCCTCCCCCCTTGTACAGCTCGTCCATGCCGAG-3'	GFP-GMA4C
Primer 3	F: 5'-CGCTGCTTCTGCACCACGCATTGTGGGGGAGGCAGTGTGAGCAAGGGCGAGGAGCT-3'	GMA4C-GFP
Primer 4	R: 5'-GCGACGAAAACCACGGCAACGGCCAGAACCATGGTGATGGTGATGGTGAGAAG-3'	GMA4C-GFP

Supplementary Table S2

Protein name	Sequence
GFP	MGSSHHHHHHGSSVSKGEELFTGVVPILVELDGDVNGHKFSVRGEGEGDATNGKLTLKFICTTGKLPVPWPTLVTTLTYGVQCFSRYPDHMKQHDFFKSAMPEGYVQERTISFKDDGTYKTRAEVKFEGDTLVNRIELKGIDFKEDGNILGHKLEYNFNSHNVYITADKQKNGIKANFKIRHNVEDGSVQLADHYQQNTPIGDGPVLLPDNHYLSTQSKLSKDPNEKRDHMVLLEFVTAAGITLGMDELYKGIEENLYFQSNIGSG
GMA4C-GFP	MGSSHHHHHHGSGRCRGFRRRCFCTTHCGGGSVSKGEELFTGVVPILVELDGDVNGHKFSVRGEGEGDATNGKLTLKFICTTGKLPVPWPTLVTTLTYGVQCFSRYPDHMKQHDFFKSAMPEGYVQERTISFKDDGTYKTRAEVKFEGDTLVNRIELKGIDFKEDGNILGHKLEYNFNSHNVYITADKQKNGIKANFKIRHNVEDGSVQLADHYQQNTPIGDGPVLLPDNHYLSTQSKLSKDPNEKRDHMVLLEFVTAAGITLGMDELYKGIEENLYFQSNIGSG
GFP-GMA4C	MGSSHHHHHHGSSVSKGEELFTGVVPILVELDGDVNGHKFSVRGEGEGDATNGKLTLKFICTTGKLPVPWPTLVTTLTYGVQCFSRYPDHMKQHDFFKSAMPEGYVQERTISFKDDGTYKTRAEVKFEGDTLVNRIELKGIDFKEDGNILGHKLEYNFNSHNVYITADKQKNGIKANFKIRHNVEDGSVQLADHYQQNTPIGDGPVLLPDNHYLSTQSKLSKDPNEKRDHMVLLEFVTAAGITLGMDELYKGGGSGRCRGFRRRCFCTTHC
GMA4C	GRCRHGFRRRCFCTTHC

Supplementary Table S3

Protein Name	Sequence of synthesized DNA
GFP	ATGGGTTCTTCTCACCATCACCATCACCATGGTTCTTCTGTGAGCAAGGGCGAGGAGCT<br>GTTCACCGGGGTGGTGCCCATCCTGGTCGAGCTGGACGGCGACGTAAACGGCCACAAG<br>TTCAGCGTGCGCGGCGAGGGCGAGGGCGATGCCACCAACGGCAAGCTGACCCTGAAG<br>TTCATCTGCACCACCGGCAAGCTGCCCGTGCCCTGGCCCACCCTCGTGACCACCCTGA<br>CCTACGGCGTGCAGTGCTTCAGCCGCTACCCCGACCACATGAAGCAGCACGACTTCTTC<br>AAGTCCGCCATGCCCGAAGGCTACGTCCAGGAGCGCACCATCTCCTTCAAGGACGACG<br>GCACCTACAAGACCCGCGCCGAGGTGAAGTTCGAGGGCGACACCCTGGTGAACCGCAT<br>CGAGCTGAAGGGCATCGACTTCAAGGAGGACGGCAACATCCTGGGGCACAAGCTGGAG<br>TACAACTTCAACAGCCACAACGTCTATATCACGGCCGACAAGCAGAAGAACGGCATCAA<br>GGCGAACTTCAAGATCCGCCACAACGTCGAGGACGGCAGCGTGCAGCTCGCCGACCAC<br>TACCAGCAGAACACCCCCATCGGCGACGGCCCCGTGCTGCTGCCCGACAACCACTACC<br>TGAGCACCCAGTCCAAGCTGAGCAAAGACCCCAACGAGAAGCGCGATCACATGGTCCT<br>GCTGGAGTTCGTGACCGCCGCCGGGATCACTCTCGGCATGGACGAGCTGTACAAGGGG<br>ATCGAGGAAAACCTGTACTTCCAATCCAATATTGGAAGTGGATAA
GMA4C - GFP	ATGGGTTCTTCTCACCATCACCATCACCATGGTTCTGGCCGTTGCCGTGGTTTTCGTCGC<br>CGCTGCTTCTGCACCACGCATTGTGGGGGAGGCAGTGTGAGCAAGGGCGAGGAGCTGT<br>TCACCGGGGTGGTGCCCATCCTGGTCGAGCTGGACGGCGACGTAAACGGCCACAAGTT<br>CAGCGTGCGCGGCGAGGGCGAGGGCGATGCCACCAACGGCAAGCTGACCCTGAAGTT<br>CATCTGCACCACCGGCAAGCTGCCCGTGCCCTGGCCCACCCTCGTGACCACCCTGACC<br>TACGGCGTGCAGTGCTTCAGCCGCTACCCCGACCACATGAAGCAGCACGACTTCTTCAA<br>GTCCGCCATGCCCGAAGGCTACGTCCAGGAGCGCACCATCTCCTTCAAGGACGACGGC<br>ACCTACAAGACCCGCGCCGAGGTGAAGTTCGAGGGCGACACCCTGGTGAACCGCATCG<br>AGCTGAAGGGCATCGACTTCAAGGAGGACGGCAACATCCTGGGGCACAAGCTGGAGTA<br>CAACTTCAACAGCCACAACGTCTATATCACGGCCGACAAGCAGAAGAACGGCATCAAGG<br>CGAACTTCAAGATCCGCCACAACGTCGAGGACGGCAGCGTGCAGCTCGCCGACCACTA<br>CCAGCAGAACACCCCCATCGGCGACGGCCCCGTGCTGCTGCCCGACAACCACTACCTG<br>AGCACCCAGTCCAAGCTGAGCAAAGACCCCAACGAGAAGCGCGATCACATGGTCCTGCT<br>GGAGTTCGTGACCGCCGCCGGGATCACTCTCGGCATGGACGAGCTGTACAAGGGGATC<br>GAGGAAAACCTGTACTTCCAATCCAATATTGGAAGTGGATAA
GFP-GMA4C	ATGGGTTCTTCTCACCATCACCATCACCATGGTTCTTCTGTGAGCAAGGGCGAGGAGCT<br>GTTCACCGGGGTGGTGCCCATCCTGGTCGAGCTGGACGGCGACGTAAACGGCCACAAG<br>TTCAGCGTGCGCGGCGAGGGCGAGGGCGATGCCACCAACGGCAAGCTGACCCTGAAG<br>TTCATCTGCACCACCGGCAAGCTGCCCGTGCCCTGGCCCACCCTCGTGACCACCCTGA<br>CCTACGGCGTGCAGTGCTTCAGCCGCTACCCCGACCACATGAAGCAGCACGACTTCTTC<br>AAGTCCGCCATGCCCGAAGGCTACGTCCAGGAGCGCACCATCTCCTTCAAGGACGACG<br>GCACCTACAAGACCCGCGCCGAGGTGAAGTTCGAGGGCGACACCCTGGTGAACCGCAT

	CGAGCTGAAGGGCATCGACTTCAAGGAGGACGGCAACATCCTGGGGCACAAGCTGGAG TACAACTTCAACAGCCACAACGTCTATATCACGGCCGACAAGCAGAAGAACGGCATCAA GGCGAACTTCAAGATCCGCCACAACGTCGAGGACGGCAGCGTGCAGCTCGCCGACCAC TACCAGCAGAACACCCCCATCGGCGACGGCCCCGTGCTGCTGCCCGACAACCACTACC TGAGCACCCAGTCCAAGCTGAGCAAAGACCCCAACGAGAAGCGCGATCACATGGTCCT GCTGGAGTTCGTGACCGCCGCCGGGATCACTCTCGGCATGGACGAGCTGTACAAGGGG GGAGGCAGTGGCCGTTGCCGTGGTTTTCGTCGCCGCTGCTTCTGCACCACGCATTGTTA A

Supplementary Figure S1

Recombinant GFP with GMA4C at either the N- or C-terminus (GMA4C-GFP or GFP-GMA4C, respectively) was cloned into the pET28 vector and expressed in *E. coli* BL21 (DE3) cells. All plasmids feature an N-terminus Hisx6 tag. Figure S1A illustrates the expressed constructs. Figure S1B shows the structural model of the proteins. Detailed protein sequences are found in Table S2.

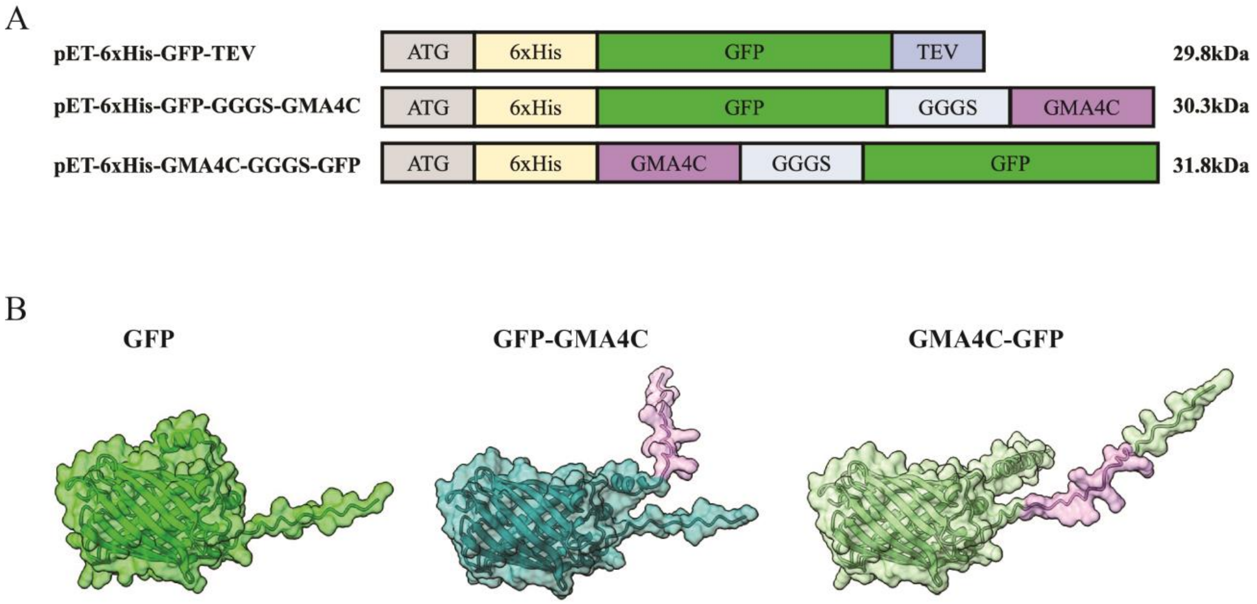

Figure S1. Expression of recombinant GFP protein with N- and C-terminus GMA4C tag. (A) Illustration of protein constructs. **(B)** Comparison of AlphaFold models of GFP-tagged proteins with the GMA4C peptide. Surface structure representations created with ChimeraX software.

Supplementary Figure S2

To assess GMA4C's capacity for cell penetration across different cell types, we subjected HGF cells to various concentrations of FITC-GMA4C. After two hours of incubation and thorough washing, we examined the cells using confocal microscopy imaging. Figure S2 presents a series of images, including representative brightfield, DAPI (blue), FITC (green), and merged images. The images show the accumulation of FITC-GMA4C within the cells.

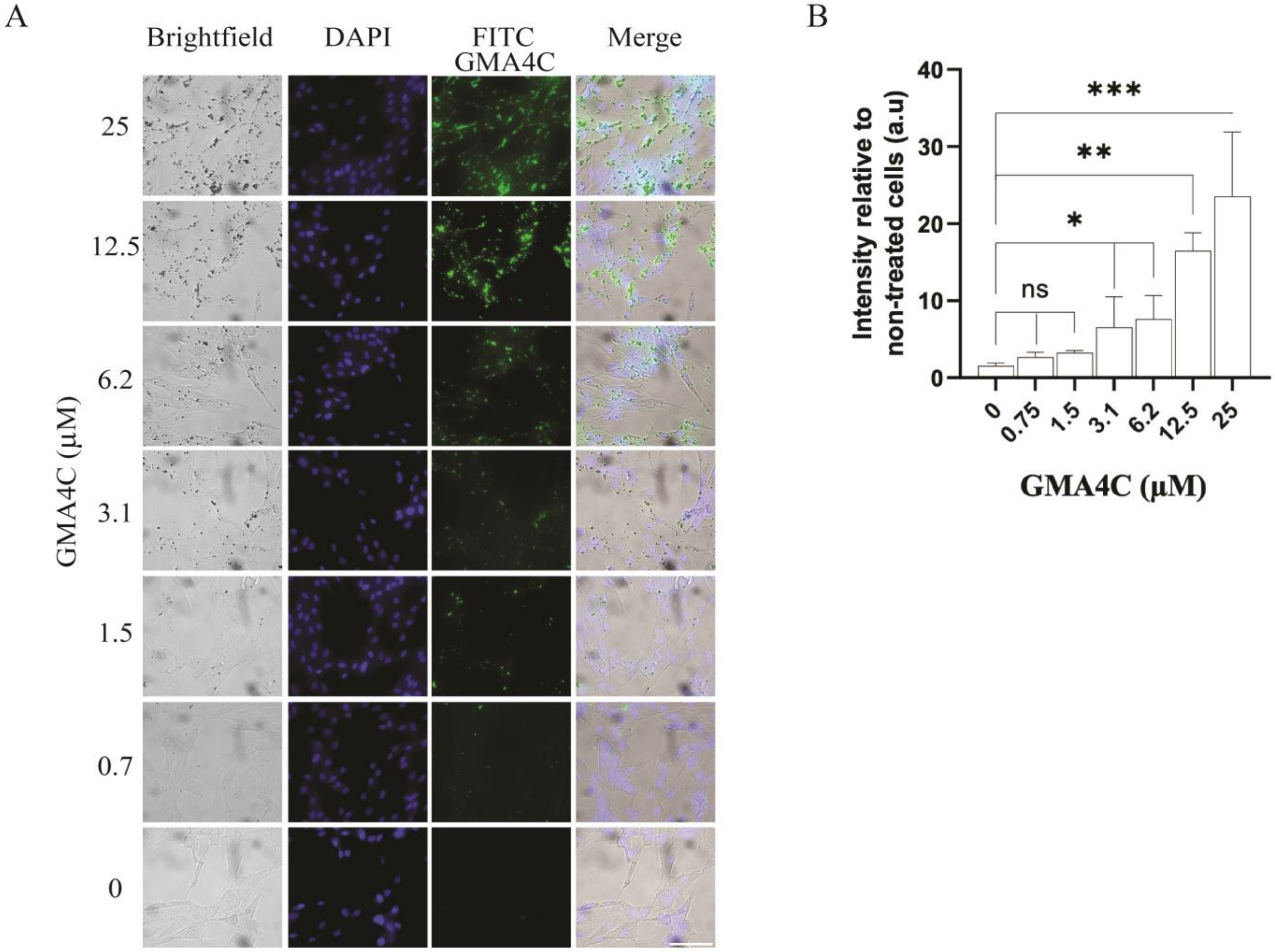

Figure S2. FITC-GMA4C Penetration into HGF Cells. (A) HGF cells were cultured for two hours with varying concentrations of FITC-GMA4C and subsequently stained with DAPI. Confocal microscopy images were captured to visualize FITC-GMA4C (green) and DAPI (blue) in cells attached to a slide. Scale bar: 100 µm. (B) Mean green fluorescence intensity relative to non-treated cells and normalized to the cell count. The signal was quantified using the Color Threshold tool in Fiji. Significance levels are denoted as follows: *p<0.05, **p<0.01, ***p<0.005.

Supplementary Figure S3

To investigate the intracellular protein uptake facilitated by the GMA4C peptide across various cell lines, we treated C2C12 myoblasts with either 5 μM of FITC-GMA4C peptide or GFP-tagged proteins for 2 hours. Subsequently, the cells underwent three PBS washes and fixation using 4% PFA. Finally, they were mounted on a glass slide for evaluation through confocal microscopy. Consistent with the results presented in Figure 3, the fluorescence microscopy images herein further confirm the efficient cellular uptake of FITC-GMA4C. Additionally, these images reveal partial colocalization with the endosomal marker Cav-1. A similar outcome was observed in HeLa cells (Figs. 5 and 6), where GMA4C effectively demonstrated its competence in internalizing GFP cargo into the cells.

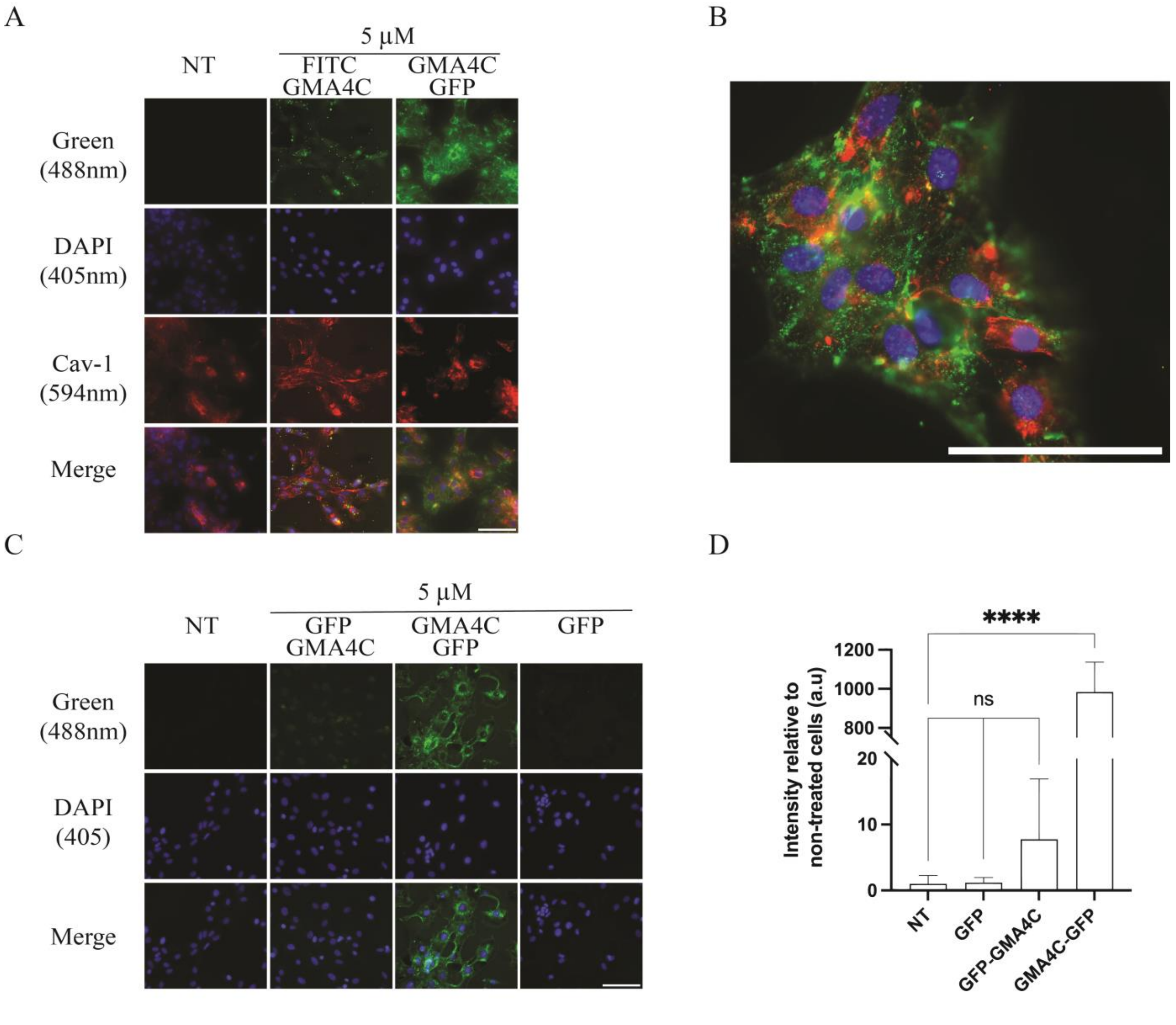

Figure S3. Facilitation of GFP Protein Delivery by GMA4C in C2C12 Cells. (A) Partial colocalization of FITC-GMA4C and GMA4C-GFP in C2C12 cells. Cells were incubated with 5 μM of FITC-GMA4C or GMA4C-GFP for two hours, fixed, mounted, and scanned by a confocal microscope. The images visualize FITC or GFP (green), DAPI (blue), and Cav-1 (red). Scale bar: 100 μm. Colocalization is indicated when similarly shaped structures appear yellow in the Merge. (B) Representative figure of GMA4C-GFP taken with a 63x Objective Lens. Scale bar: 100 μm. (C) Confocal microscopy images were captured to visualize GFP tagged with the GMA4C peptide (green) and DAPI (blue) in cells attached to a slide. Scale bar: 100 μm. (D) Mean green fluorescence intensity relative to non-treated cells and normalized to cell count. Measurement values were obtained using the Color Threshold tool in Fiji.

Supplementary Figure S4

To assess the cellular uptake of FITC-GMA4C, we incubated fungal cells, *A. flavus* with varying concentrations of FITC-GMA4C. Figure S4 displays representative images, including brightfield, DAPI (nuclear DNA stain), green fluorescence (FITC), and merged images. Notably, these images reveal the accumulation of FITC-GMA4C within the cells.

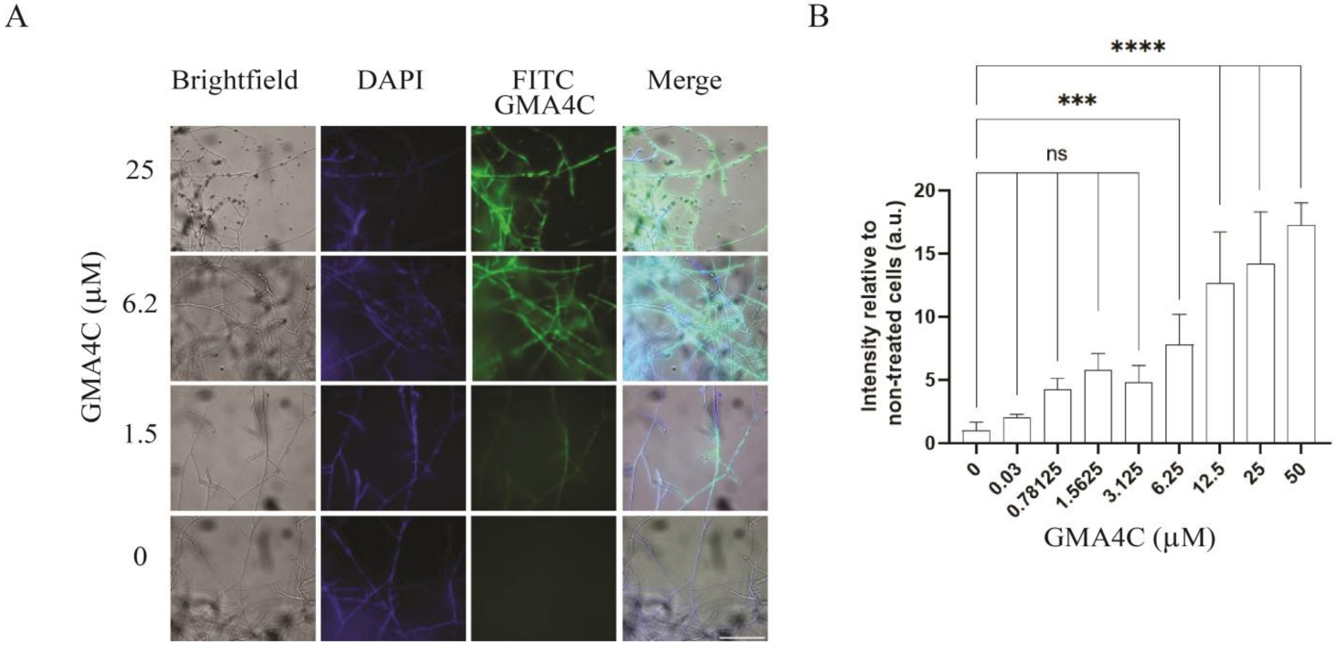

Figure S4. FITC-GMA4C penetrates into *A. flavus*. (A) Fungal cells were cultured for two hours with variable concentrations of FITC-GMA4C and stained with DAPI. Images of cells attached to a glass slide were taken by confocal microscopy visualizing FITC-GMA4C (green) and DAPI (Blue). Scale bar: 100µm. **(B)** Mean green fluorescence intensity relative to non-treated cells and normalized to the number of cells. Values were measured using the Color Threshold tab in Fiji. *:$p<0.05$, **:$p<0.01$, ***:$p<0.005$.

A. flavus culture and imaging

A.flavus A11 strain were generously provided by the laboratory of Prof. Nir Osherov. Spores were initially seeded on YAG-agarose 10 cm plates and allowed to grow for 3-4 days in a humidified incubator at 30 °C. After sporulation, fresh spores were collected using 5 ml of double-distilled water (DDW) containing 0.2% Tween. The spores were then centrifuged at room temperature for 5 minutes at 4,000 RPM and suspended in 5 ml of DDW to create the *A.flavus* fresh stock. To determine the spore concentration in the fresh stock, a 1,000-fold dilution was made with DDW, and the spores were counted using a cytometer. For microscopy purposes, 100 µL of fungal spores were cultured in 10 mL of fungal media (consisting of RPMI-1640 Medium, 100 U/mL penicillin, 100 mg/mL streptomycin, and 165 mM MOPS) in a 10 cm round dish for overnight hyphal growth at 30 °C. The following day, hyphae were collected, resuspended, and cultured with 1 mL of fungal media on top of round-glass coverslips placed in a 24-well plate. Fungi were then incubated for 16 hours at 30 °C. Subsequently, the fungal hyphae were

treated with the indicated concentrations of FITC-GMA4C peptide and incubated for 2 hours at 30°C. The treated fungus was washed three times with cold PBS, followed by gentle centrifugation at 4,000 rpm for 10 minutes. Finally, the fungus was fixed with 4% paraformaldehyde (PFA), washed with PBS, and examined using confocal microscopy imaging.

Supplementary Figure S5

To explore a potential mechanism for the cellular entry of GMA4C-GFP and GFP-GMA4C, we conducted an experiment in which HeLa cells were incubated with the proteins for two hours at 37 ℃ and 4 ℃. Our aim was to assess the extent of permeabilization of the proteins and investigate the impact of temperature on cellular permeability. To quantify the levels of permeabilized proteins, we performed a flow cytometry analysis on HeLa cells exposed to varying temperature treatments, enabling us to compare their effects on cellular permeability.

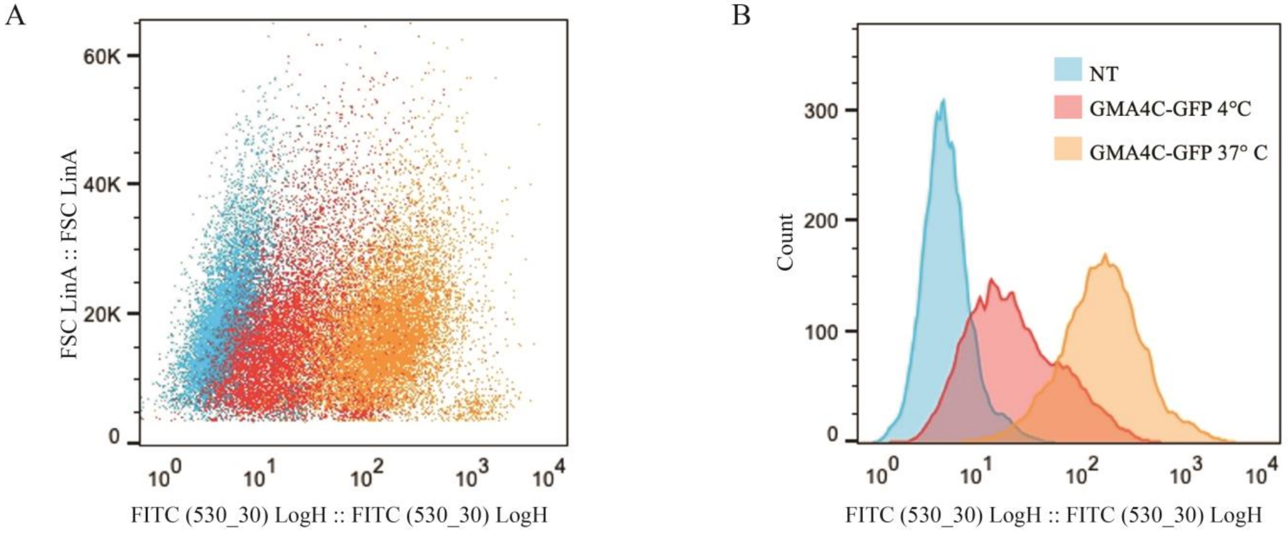

Figure S5: Flow Cytometry Analysis of HeLa cells incubated in different temperatures. HeLa cells were incubated with GMA4C-GFP recombinant proteins at a concentration of 5 μM for two hours at 37 ℃ and 4 ℃. Subsequently, the cells were washed with PBS and trypsinized. The cells were then resuspended in PBS supplemented with 2.5% FBS. **(A)** The Y-axis represents forward scatter size (FSC), where an increased signal indicates an increase in cell size or budding. The X-axis represents the GFP signal in different cell populations. **(B)** The histogram displays the count of cells versus GFP intensity for each treatment.

Supplementary Figure S6

To quantify the extent of GMA4C-GFP penetration into cells, HeLa cells were incubated with variable protein concentrations for two hours at 37°C. Subsequently, the cells were washed with PBS, fixed, and stained with DAPI (blue) and β-tubulin (red) for visualization using confocal imaging (Fig. S6-A). To assess the percentage of cellular permeability, we utilized a threshold module in Fiji software to quantify the green and red channels. The selected green areas were then divided by the total cellular surface area indicated by the red channel (β-tubulin). These values were normalized relative to untreated cells' mean green/red fluorescence intensity ratio, revealing a dose-dependent signal for GMA4C-GFP penetration into the cells (Fig. S6-B). Furthermore, we calculated the percentage of cells penetrated by GMA4C-GFP by determining Pearson's coefficient, which provides a statistical measure of the linear relationship between the green and red channels at different GMA4C-GFP concentrations (Fig. S6-C). Notably, a clear correlation is observed, indicating a penetration rate of nearly 100% in cells treated with 10 µM of the protein.

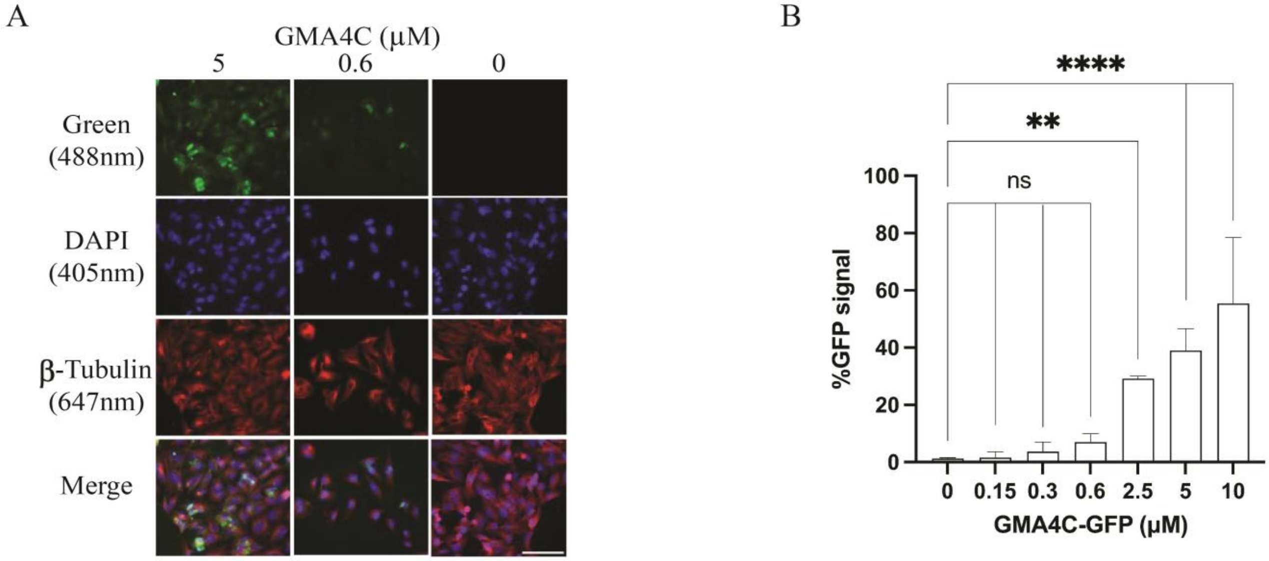

C

GMA4C-GFP (µM)	% Cellular uptake	Pearson coefficient
10	99.3	0.52±0.14
5	90.7	0.39
2.5	19.02	0.35
0.3	0	0.335±0.06
0	0	0.095±0.007

Figure S6. Cellular Delivery of GMA4C-GFP in HeLa Cells. (A) HeLa cells were incubated with variable concentrations of GMA4C-GFP for 2 hours. Cells were subsequently stained and imaged for DAPI (blue), GMA4C-GFP (green), and β-tubulin (red), and the cellular uptake of the protein was analyzed. Representative fields were imaged at 40× magnification. Scale bar: 100 µm. **(B)** Quantification of confocal image signal intensity. The bars represent the mean green fluorescence intensity relative to non-treated cells, normalized to the cellular surface area measured by β-Tubulin. Values were measured using the Color Threshold tool in Fiji. The values represent the mean ± SD of four replicates. **(C)** Measurement of protein penetration levels. The table summarizes Pearson's coefficient and the cellular uptake percentage of GMA4C-GFP. The latter was calculated based on the number of green pixels that colocalized with the cellular margins of red pixels (tubulin) and were above the threshold in both channels. The Pearson's coefficient for channel-1 (green) vs. channel-2 (red) was measured using the "Color 2" plugin in Fiji and is presented as the mean ± SD of duplicates.

Supplementary Figure S7

To assess the protein delivery capability of GMA4C into cells without slide fixation, we utilized live-cell imaging and Western blot analysis. HeLa cells were grown in 96-well glass-bottom plates or 10-cm dishes and treated with media containing the specified concentrations of FITC-GMA4C and GMA4C-GFP (Fig. S7A and S7B, respectively) and DAPI at 37°C in a 5% CO_2 atmosphere. Subsequently, the cells were washed with PBS and provided with fresh media for imaging.

For Western blotting, HeLa cells were supplemented with 5 µM of GFP and GMA4C-GFP for two hours, then washed with PBS, scraped, and subjected to sonication twice in Buffer H (see methods) with a 1:200 dilution of Protease Inhibitor (cat. 539134, Merck). Cell lysates were prepared with 4x sample buffer and resolved on a 10% SDS-PAGE gel. The signal was detected using specific antibodies after incubation overnight with primary antibodies. Figure S7C presents Western blots from HeLa cells treated with GFP and GMA4C-GFP. In all samples treated with either GFP or GMA4C-GFP, a distinct, intense fluorescent band was observed in the medium fraction. Furthermore, a shift in band size was noted between GFP and GMA4C-GFP, corresponding to their molecular weights (29.8 kDa and 31.8 kDa, respectively). A band showing the penetration of GMA4C-GFP to the cells is observed.

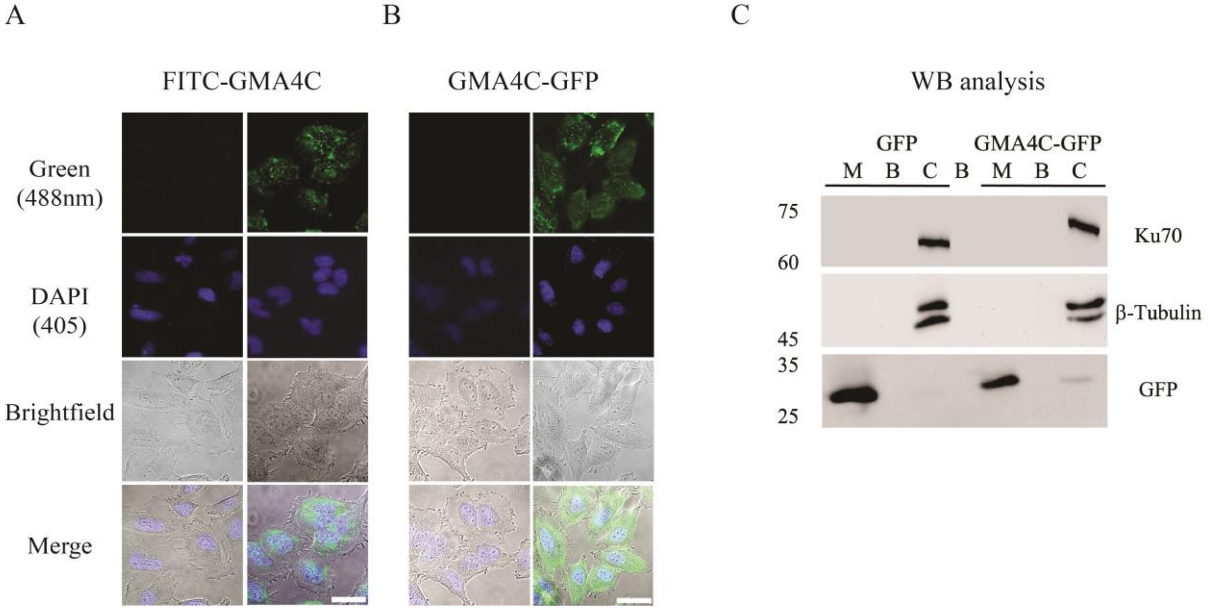

Figure S7. Live cell imaging and WB analysis of HeLa cells. Cells were treated with **(A)** FITC-GMA4C or **(B)** GMA4C-GFP along with DAPI for 2 hrs. Images were taken without cellular fixation using a confocal microscope with Differential Interference Contrast (brightfield) and a 488 nm laser. 20x lenses were used. Scale bar: 40 µm. **(C)** HeLa cells were cultured for two hours with GFP or GMA4C-GFP, then were immunoblotted for Ku70, β-tubulin and GFP. M - Medium, B - blank lane, C- Cells.

Supplementary Figure S8

Full membrane Blot images of Fig. S7C.

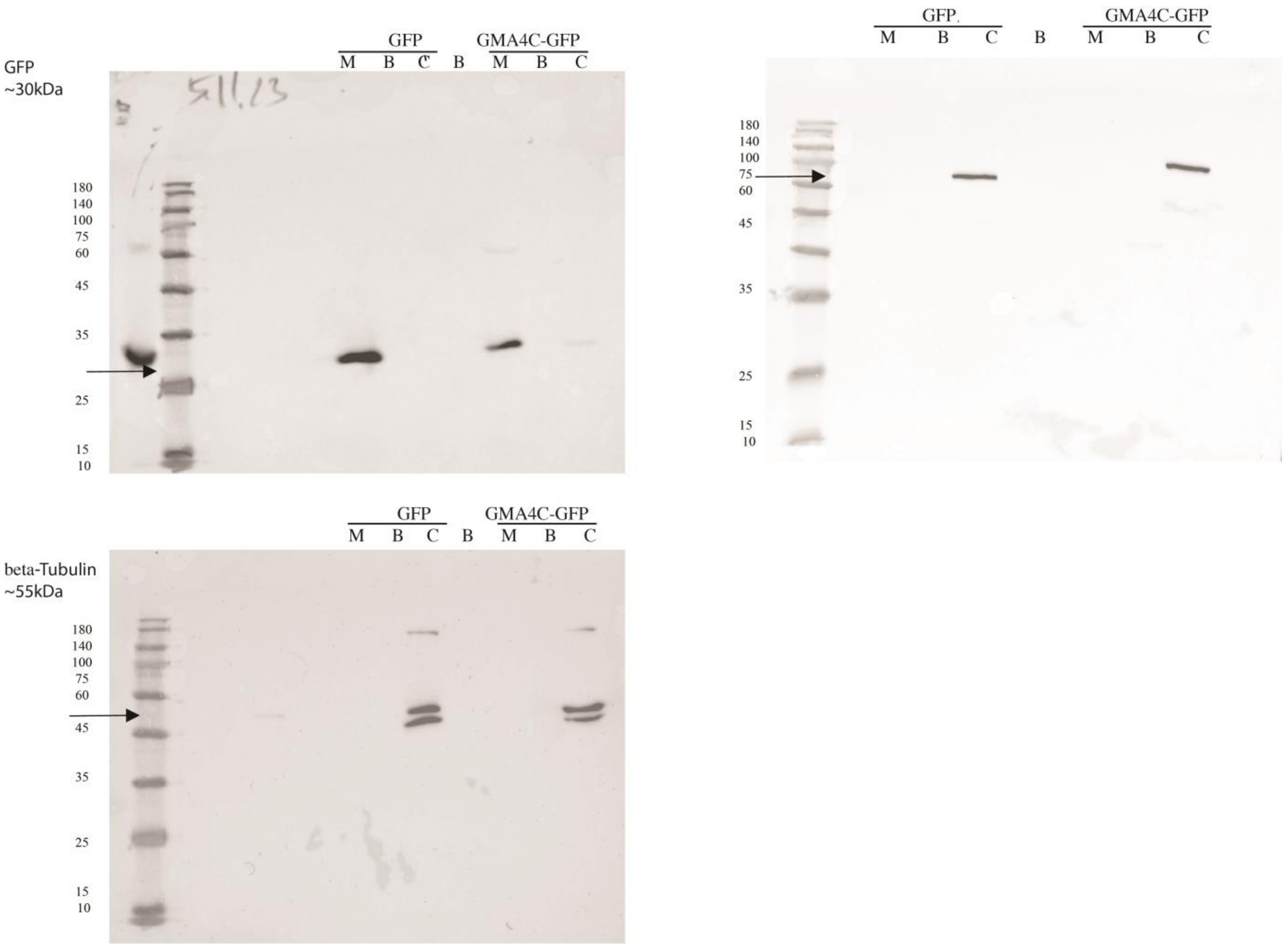

Figure S8. WB analysis of HeLa cells showing the full membrane. HeLa cells were cultured for two hours with GFP or GMA4C-GFP, then were immunoblotted for Ku70, β-tubulin and GFP. M - Medium, B - blank lane, C- Cells.
